# The Influence of Anthropometrics on Cardiac Mechanics in Healthy Women With Opposite Obesity Phenotypes (Android vs Gynoid)

**DOI:** 10.7759/cureus.51698

**Published:** 2024-01-05

**Authors:** Andrea Sonaglioni, Anna Ferrulli, Gian Luigi Nicolosi, Michele Lombardo, Livio Luzi

**Affiliations:** 1 Cardiology, Istituto di Ricovero e Cura a Carattere Scientifico (IRCCS) MultiMedica, Milan, ITA; 2 Endocrinology, Diabetes and Metabolism, Istituto di Ricovero e Cura a Carattere Scientifico (IRCCS) MultiMedica, Milan, ITA; 3 Cardiology, Policlinico San Giorgio, Pordenone, ITA

**Keywords:** modified haller index, subclinical myocardial dysfunction, whr, gynoid obesity, android obesity

## Abstract

Background: The possible influence exerted by mechanical factors and/or compressive phenomena on myocardial strain parameters in healthy individuals with opposite obesity phenotypes (android vs gynoid) has never been previously investigated. Accordingly, we aimed at evaluating the relationship between anthropometrics, such as the waist-to-hip ratio (WHR), modified Haller index (MHI, the ratio of chest transverse diameter over the distance between sternum and spine), and epicardial adipose tissue (EAT), and left ventricular (LV)-global longitudinal strain (GLS), in healthy women with opposite obesity phenotypes (android vs gynoid).

Methods: Forty healthy women with obesity (body mass index (BMI) ≥30 Kg/m^2)^ and WHR ≥0.85 (“android group”) (52.5±13.2 yrs), 40 age- and BMI-matched healthy women with obesityand WHR <0.78 (“gynoid group”) (49.8±13.4 yrs) and 40 age-matched healthy women without obesity (BMI <30 Kg/m^2^) (controls) (50.3±12.5 yrs) were retrospectively analyzed. All women underwent transthoracic echocardiography implemented with echocardiographic strain analysis of all cardiac chambers. Correlation between LV-GLS and anthropometrics (WHR, MHI, and EAT) was assessed in both groups of obese women. Age, WHR, homeostasis model assessment for insulin resistance (HOMA-IR), and left ventricular mass index (LVMi) were included in the logistic regression analysis performed for evaluating the independent predictors of reduced LV-GLS magnitude (less negative than -20%) in women with android obesity.

Results: Compared to the other groups of women, those with android obesity were found with significantly greater LVMi, higher LV filling pressures, and lower biventricular and biatrial deformation indices. A strong inverse correlation between LV-GLS and all anthropometrics (WHR, MHI, and EAT) was demonstrated in both groups of women with obesity. Univariate logistic regression analysis revealed that WHR (OR 1.58, 95%CI 1.22-2.03, p<0.001) and LVMi (OR 1.09, 95%CI 1.02-1.16, p=0.006) were independently correlated with LV-GLS impairment in women with android obesity. On multivariate logistic regression analysis, the WHR maintained a statistically significant association with the above-mentioned outcome (OR 1.68, 95%CI 1.14-2.48, p=0.009). Receiver operating characteristic (ROC) curve analysis showed that a WHR value ≥1.01 had 93% sensitivity and 100% specificity for detecting LV-GLS impairment in women with android obesity (AUC=0.98; 95%CI 0.96-1.00).

Conclusions: Anthropometrics may strongly influence cardiac mechanics in healthy women with obesity. The WHR is associated with reduced LV-GLS magnitude in healthy women with android obesity, independent of age, glycometabolic status, and LV size.

## Introduction

During the last few decades, the prevalence of obesity has increased worldwide, particularly in American and European countries, mainly due to the adoption of a sedentary lifestyle and the decline in overall physical activity in combination with the consumption of unhealthy diets [1]. This condition carries a significant risk for unfavorable ventricular remodeling and subsequent heart failure (HF) development [2].

Recently, particular attention has been given to patterns of body fat distribution and their impact on left ventricular (LV) morphology and function. Recent studies [3-5] have demonstrated that abdominal obesity and increased visceral adiposity are associated with decreased LV and right ventricular (RV) mechanics, early detected by two-dimensional (2D) speckle tracking echocardiography (STE), an angle-independent technique, which provides incremental diagnostic and prognostic information over conventional transthoracic echocardiography (TTE). On the other hand, studies conducted on individuals with gynoid obesity [6-8] have demonstrated that lower-body fat accumulation, which is much more common in women than in men [9], is inversely associated with cardiovascular disease and glycometabolic complications.

The association between central obesity and subclinical ventricular dysfunction has been explained by a number of pathophysiological mechanisms, such as a chronic inflammatory state [10], the development of insulin resistance [11], the activation of the renin-angiotensin system [12], alterations in lipid metabolism causing increased arterial stiffness [13], and finally impaired coronary microcirculation [14], all induced by the increased visceral fat, resulting in ventricular dysfunction.

As far as we know, no previous study evaluated the possible influence exerted by mechanical factors and/or compressive phenomena on myocardial strain parameters in healthy individuals with opposite obesity phenotypes (android vs gynoid). We hypothesized that the combination of abdominal obesity, measured by the waist-to-hip ratio (WHR), and both external and internal thoracic adiposity, measured by modified Haller index (MHI, the ratio of chest transverse diameter over the distance between sternum and spine) [15] and epicardial adipose tissue (EAT) thickness respectively, might synergically contribute to a significant reduction in LV global longitudinal strain (GLS) magnitude in individuals with android obesity and, conversely, that, in individuals with the accumulation of adipose tissue in the gluteal-femoral regions, such as women with gynoid obesity, these compressive phenomena are significantly less pronounced or totally absent. Accordingly, the present study was primarily designed to accurately define cardiac kinetics and function by conventional 2D-TTE implemented with echocardiographic strain imaging in two retrospective cohorts of healthy women with opposite obesity phenotypes (android vs gynoid) and to evaluate the effect of increased abdominal obesity and thoracic adiposity on LV-GLS magnitude.

## Materials and methods

Study population

The present study was a retrospective observational study that analyzed two cohorts of healthy women with android and gynoid obesity matched by age and body mass index (BMI) compared to a control group of healthy individuals without obesity, who underwent a conventional 2D-TTE implemented with 2D-STE analysis of all cardiac chambers, between September 2017 and April 2022. The three groups of healthy women were selected from a larger study population included in the two MHI validation studies [15,16].

Android obesity was defined by BMI ≥30 Kg/m^2^ and WHR ≥0.85, whereas gynoid obesity was defined by BMI ≥30 Kg/m^2^ and WHR <0.78 [17]. The control group was composed of healthy women without obesity (BMI <30 Kg/m^2^) matched by age and cardiovascular risk factors, as per European Society of Cardiology guidelines [18].

The healthy status in women with obesity was defined by the absence of symptoms, the evidence of normal findings on physical examination and resting electrocardiogram (ECG), and the absence of overt heart disease on conventional TTE.

Criteria of exclusion were the following: male sex; WHR between 0.78 and 0.84; hypertension, defined as a systolic blood pressure (SBP) ≥140 mmHg and/or a diastolic blood pressure (DBP) ≥90 mmHg or the use of antihypertensive medications; type 2 diabetes mellitus, defined as a fasting plasma glucose level of more than 126 mg/dl or the use of hypoglycemic medication or a self-reported history; atrial fibrillation; left bundle branch block on resting ECG; history of coronary artery disease (previous myocardial infarction, previous percutaneous coronary intervention or previous coronary artery bypass graft); moderate-to-severe mitral and/or aortic valve disease; hypertrophic, infiltrative and/or dilated cardiomyopathy; history of congenital heart disease; left ventricular ejection fraction (LVEF) ≤55%; acute coronary syndrome, acute congestive heart failure, acute respiratory failure, acute and chronic renal failure (defined as an estimated glomerular filtration rate <60 ml/min/m2 [19]); hemodynamic instability; poor echocardiographic acoustic windows (not adequate for appropriate visualization and definition of endocardial border of all cardiac chambers).

During the same day, each participant underwent accurate anamnesis, physical examination, blood pressure measurement, anthropometric measurements (BMI, WHR, and MHI), blood tests, and conventional 2D-TTE implemented with 2D-STE analysis of all cardiac chambers.

All procedures were performed according to the ethical standards of the institutional research committee and to the Declaration of Helsinki (1964) and its subsequent amendments or equivalent ethical standards. Written informed consent was obtained from all participants.

WHR measurement

Waist and hip circumferences were measured using a flexible narrow non-stretch tape while the participants were standing and wearing loose-fitting clothing. Waist circumference was measured halfway between the lower ribs and the iliac crest, while hip circumference was measured at the largest circumference around the buttocks [17].

Modiﬁed Haller index calculation

The modified Haller index was calculated by dividing the maximum latero-lateral (L-L) external thoracic diameter (using a measuring device graduated in centimeters) by the minimum antero-posterior (A-P) internal thoracic diameter (measured in centimeters from the focused 2D-TTE) [15].

Biochemical and glycometabolic parameters

Blood tests measured serum levels of creatinine, total cholesterol, high-density lipoprotein (HDL) cholesterol, triglycerides, low-density lipoprotein (LDL) cholesterol, fasting glucose, glycosylated hemoglobin, fasting insulin and finally the homeostasis model assessment for insulin resistance (HOMA-IR), calculated according to the formula: fasting insulin (microU/L) x fasting glucose (mg/dl)/405 [20].

Conventional echoDoppler examination

All echocardiograms were performed by using a Philips Sparq ultrasound machine (Philips, Andover, Massachusetts, USA) with a 2.5 MHz transducer. All parameters were measured according to the Recommendations of the American Society of Echocardiography and the European Association of Cardiovascular Imaging [21,22].

Following conventional 2D echoDoppler parameters were recorded: relative wall thickness (RWT), left ventricular mass index (LVMi) calculated by the Devereux’s formula, left ventricular end-diastolic volume index (LVEDVi), left ventricular end-systolic volume index (LVESVi), left atrial volume index (LAVi) and left ventricular ejection fraction (LVEF) measured by the biplane modified Simpson's method [21], mitral annular plane systolic excursion (MAPSE), left ventricular diastolic function assessed by the E/A ratio and E/average e’ ratio [22], right ventricular inflow tract (RVIT), RV longitudinal function measured by tricuspid annular plane systolic excursion (TAPSE) and finally systolic pulmonary artery pressure (SPAP) calculated by the Bernoulli equation, where SPAP = 4 x tricuspid regurgitation velocity (TRV)2 + right atrial pressure; the latter was estimated by both the inferior vena cava diameter and its inspiratory collapse [23].

Measurement of the epicardial adipose tissue thickness

The epicardial adipose tissue thickness was measured at the end of systole on the free wall of the right ventricle from the parasternal long-axis view on standard 2D-TTE and was deﬁned as an echo-free or hypoechoic area adjacent to the right ventricle [24].

Hemodynamic indices

The following hemodynamic parameters were assessed: heart rate, SBP, DBP, and mean arterial pressure (MAP). The latter was calculated by the following formula: MAP = DBP + [(SBP-DBP/3)]. End-systolic pressure (ESP) was estimated as 0.9 x brachial systolic blood pressure [25]. Stroke volume (SV) was obtained from the product of the left ventricular outﬂow tract (LVOT) area and LVOT time velocity integral, using pulsed Doppler echocardiography; cardiac output (CO) was calculated by multiplying the SV by the heart rate. The effective arterial elastance index (EaI) was calculated as the ratio ESP/SVi ratio [26]. Finally, total peripheral resistance (TPR) (dyne x sec/cm5) was measured with the formula TPR = MAP (kPa)/CO (L/min) x 80 [27].

Speckle-tracking echocardiography

Immediately after conventional 2D-TTE, high-resolution 2D speckle-tracking strain analyses were performed on 2D images in the following order: apical four-chamber, two-chamber, and three-chamber views for LV longitudinal strain, and on basal, medial, and apical short-axis views for LV circumferential and radial strain, using the automated function imaging and the Q-Analysis module.

According to Philips QLAB software, the LV wall was divided into seven segments in each apical view. LV peak systolic strain was deﬁned as the point of maximal systolic shortening for longitudinal and circumferential strain, and maximal systolic thickening for radial strain, respectively. A single bull’s-eye summary for LV-GLS, LV global circumferential strain (GCS), and LV global radial strain (GRS) was obtained, presenting the analysis for each segment along with the assessment of global LV strain. The early peak diastolic strain rate was obtained from longitudinal, circumferential, and radial measurements.

RV-GLS was measured from the average of segmental strain curves obtained from the apical four-chamber view. RV free wall longitudinal strain was calculated as the mean of RV lateral basal, mid, and apical segments, with the exclusion of septal segments [28].

To calculate left atrial (LA) strain, the Philips QLAB software automatically divided the atrial wall into seven segments, and a “biplane method’ was employed. The following measurements were performed: positive global atrial strain (GSA+), during the reservoir phase; negative global atrial strain (GSA-), during the LA systole; total global atrial strain (TGSA): the sum of the two peaks. Mean GSA+, GSA-, and TGSA were calculated by averaging the four-chamber and two-chamber endocardial LA longitudinal strains. From the 2D atrial strain, strain rate curves were derived, which allowed the measurement of the atrial strain rate during the three phases: the ﬁrst positive global strain rate, from the beginning of ventricular systole; global early-diastolic strain rate; global late-diastolic strain rate. Moreover, we calculated an echocardiographic index of left atrial stiffness, that is the E/e’/GSA+ ratio [29].

Finally, for right atrial (RA) reservoir strain assessment, markers were placed on the edges of the tricuspid annulus and the endocardial side of the superior right atrial region [30].

Statistical analysis

The primary endpoint of the study was to accurately deﬁne biventricular and biatrial myocardial strain and strain rate parameters assessed by 2D-STE analysis in healthy women with android obesity and to compare these data to those obtained from women with gynoid obesity. The secondary endpoint was to determine the independent predictors of impaired LV-GLS, deﬁned as an absolute value less negative than -20% [31], in women with android obesity.

Three groups of women were analyzed: those with android obesity (Group 1), those with gynoid obesity (Group 2), and healthy women without obesity, as controls (Group 3). Each continuous variable was checked through the Shapiro-Wilk test and all data were determined to be normally distributed. Accordingly, for each group of women, continuous data are summarized as mean ± standard deviation, whereas categorical data are presented as frequency and percentage. Continuous variables were compared using the one-way analysis of variance (ANOVA), while the chi‐square test was used to compare categorical variables.

The correlation between LV-GLS and the following anthropometrics, WHR, MHI, and EAT, was evaluated separately in women with android obesity and those with gynoid obesity, by using Pearson’s correlation coefﬁcient.

Univariate logistic regression analysis was performed to evaluate the effect of the main demographic, anthropometric, glycometabolic, and conventional echocardiographic variables on LV-GLS impairment (deﬁned as an absolute value less negative than -20%) in women with android obesity. According to the one-in-ten rule (one predictive variable for every ten events), only the following variables were included in the logistic regression analysis: age (as a demographic variable), WHR (as an anthropometric variable), HOMA-IR (as a glycometabolic variable) and LVMi (as a conventional echocardiographic variable). For each variable investigated, correspondent odds ratios with 95% conﬁdence intervals (CI) were calculated. Variables with a p value <0.05 were then entered into a multivariate model.

The receiver operating characteristic (ROC) curve analysis was performed to establish the sensitivity and the speciﬁcity of the main statistically significant continuous variable for predicting an impaired LV-GLS in women with android obesity. The area under the curve (AUC) was estimated.

Statistical power analysis was conducted for this study. A sample size of 40 women with android obesity and 40 women with gynoid obesity reached 80% statistical power to detect a 2.2 points difference in the LV-GLS measured in the two groups of women assuming a pooled standard deviation of 3.5 units, using a two-sided equal-variance t-test with a level of signiﬁcance (alpha) of 5%.

A detailed intra-observer and inter-observer variability analysis for the assessment of LV-GLS was conducted in a subgroup of 10 randomly selected women with android obesity. The LV-GLS was remeasured by the cardiologist who performed all echocardiographic examinations (A.S.) and by a second one (M.L.). The analyses were performed in a blinded manner. Both raters chose the frame on which to perform each measurement. The intraclass correlation coefﬁcient (ICC) with its 95% CI was used as a statistical method for assessing intra-observer and inter-observer measurement variability. An ICC of 0.70 or more was considered to indicate acceptable reliability.

Values of p <0.05 were considered statistically significant. Statistical analysis was performed with IBM SPSS Statistics for Windows, Version 28 (Released 2021; IBM Corp., Armonk, New York, United States).

## Results

No woman with obesity was excluded due to poor echocardiographic windows and transthoracic echocardiography implemented with complete assessment of all biventricular and biatrial myocardial strain indices was performed in both groups of obese women.

A total of 40 healthy women with android obesity (mean age 52.5±13.2 yrs), 40 age- and BMI-matched healthy women with gynoid obesity (mean age 49.8±13.4 yrs), and 40 age-matched healthy controls without obesity (mean age 50.3±12.5 yrs) were retrospectively analyzed.

Table 1 summarizes all demographics, anthropometrics, and biochemical parameters measured in the three groups of women. Analysis of MHI components revealed that L-L thoracic diameter was significantly larger in healthy women with android obesity in comparison to those with gynoid obesity and controls (p <0.001); on the other hand, the A-P thoracic diameter was similar in the two groups of women with obesity, but significantly larger than controls (p <0.001). The resultant MHI value was significantly lower in women with gynoid obesity than in the other two groups of women (p <0.001), thus suggesting a more circular transversal thoracic shape in obese women with lower-body fat distribution. With regard to the blood lipid profile, women with central obesity were found with significantly lower serum levels of HDL cholesterol and significantly higher serum levels of triglycerides in comparison to women with gynoid obesity and controls, whereas serum levels of total cholesterol and LDL cholesterol were similar in the three groups of women. No woman was in statin therapy. Glycometabolic parameter assessment showed that serum levels of fasting glucose, glycosylated hemoglobin, insulin, and HOMA-IR were all significantly higher in women with android obesity than the other two groups of women (all p <0.001).

**Table 1:** Demographic, anthropometric, biochemical, and glycometabolic parameters detected in the two groups of 40 healthy women with “apple-shaped” and “pear-shaped” obesity and in the 40 controls, enrolled in the present study. Data are expressed as mean ± SD or as number (percentage). Significant p-values are in bold. A-P, antero-posterior; BMI, body mass index; BSA, body surface area; HDL, high-density lipoprotein; HOMA-IR, Homeostasis Model Assessment for Insulin Resistance; LDL, low-density lipoprotein; L-L, latero-lateral; MHI, modified Haller index; WHR, waist-to-hip ratio.

	Android obesity (n=40)	Gynoid obesity (n=40)	Controls (n=40)	p-value
Demographics				
Age (yrs)	52.5 ± 13.2	49.8 ± 13.4	50.3 ± 12.5	0.62
Anthropometrics				
Height (cm)	165 ± 0.07	165 ± 0.09	167 ± 0.08	<0.001
Weight (Kg)	100.2 ± 11.5	96.9 ± 12.5	65.8 ± 13.6	<0.001
BSA (m^2^)	2.08 ± 0.20	2.03 ± 0.18	1.73 ± 0.14	<0.001
BMI (Kg/m^2^)	36.8 ± 7.1	36.0 ± 6.0	24.0 ± 2.8	<0.001
Waist circumference (cm)	114.5 ± 15.2	95.1 ± 5.1	78.2 ± 4.5	<0.001
Hip (cm)	117.2 ± 14.3	129.0 ± 9.4	98.3 ± 7.7	<0.001
WHR	0.98 ± 0.07	0.74 ± 0.04	0.79 ± 0.03	<0.001
L-L thoracic diameter (cm)	36.1 ± 3.9	31.0 ± 3.8	30.1 ± 2.1	<0.001
A-P thoracic diameter (cm)	15.8 ± 0.8	15.7 ± 0.8	13.3 ± 0.9	<0.001
MHI	2.30 ± 0.22	1.98 ± 0.28	2.26 ± 0.16	<0.001
Biochemical and glycometabolic parameters				
Serum creatinine (mg/dl)	0.71 ± 0.12	0.70 ± 0.08	0.66 ± 0.14	0.13
Serum total cholesterol (mg/dl)	181.7 ± 37.0	182.3 ± 23.5	183.3 ± 22.1	0.97
Serum HDL cholesterol (mg/dl)	41.8 ± 9.0	62.2 ± 7.5	60.6 ± 6.5	<0.001
Serum LDL cholesterol (mg/dl)	115.3 ± 28.9	107.1 ± 24.4	105.1 ± 21.2	0.16
Serum total triglycerides (mg/dl)	144.9 ± 68.5	95.8 ± 24.2	90.4 ± 22.6	<0.001
Fasting serum glucose (mg/dl)	115.5 ± 19.5	92.5 ± 9.2	82.8 ± 10.6	<0.001
Serum glycosylated hemoglobin (mmol/mol)	42.1 ± 7.3	35.0 ± 3.6	32.2 ± 4.4	<0.001
Fasting serum insulin (mU/ml)	21.0 ± 9.1	7.2 ± 2.2	3.8 ± 2.1	<0.001
HOMA-IR	6.1 ± 3.3	1.6 ± 0.5	0.78 ± 0.4	<0.001

On conventional echocardiographic examination, women with android obesity were diagnosed with significantly greater RWT and LVMi in comparison to the women with gynoid obesity and controls (both p <0.001). LV concentric remodeling was the most common LV geometric pattern detected in women with central obesity, being observed in approximately half of cases. Conversely, women with gynoid obesity were more frequently diagnosed with a normal LV geometric pattern (60% of cases) or with LV eccentric hypertrophy (20% of cases). Interestingly, the indexed LV end-diastolic and end-systolic volumes measured in women with android obesity were significantly smaller in comparison to those measured in the other groups of women. This finding might suggest compressive phenomena on LV mechanics in women with central obesity. Biventricular systolic function was normal in all groups of women. However, absolute values of MAPSE and TAPSE, expression of biventricular longitudinal function, were significantly lower in women with abdominal obesity than in those with gynoid obesity and controls (both p <0.001). Concerning LV diastolic function, an impaired relaxation pattern (first degree of diastolic dysfunction) was much more commonly detected in women with android obesity and controls than in those with gynoid obesity (p <0.001). The latter were much more frequently diagnosed with a normal LV transmitral flow pattern together with normal LV filling pressures, as non-invasively expressed by the E/average e’ ratio, in comparison to women with central obesity and controls. As a consequence of LV concentric remodeling associated with increased LV filling pressures, women with android obesity were found with significantly larger left atrial end-systolic dimensions than the other groups of women. No significant valvulopathy was observed in the three study groups of obese women, whereas ascending thoracic aorta size, measured from the aortic root to the aortic arch, was significantly larger in women with central obesity compared to the other groups of women (all p <0.001). Finally, as expected, the end-systolic epicardial fat thickness was significantly greater in women with android obesity (Table 2).

**Table 2:** Conventional echoDoppler parameters and hemodynamic indices measured in the two groups of 40 healthy women with “apple-shaped” and “pear-shaped” obesity and in the 40 controls, enrolled in the present study. Data are expressed as mean ± SD or as number (percentage). Significant P values are in bold. A-P, antero-posterior; AR, aortic regurgitation; COi, cardiac output index; DBP, diastolic blood pressure; EaI, arterial elastance index; EAT, epicardial adipose tissue; ESP, end-systolic pressure; HR, heart rate; IVS, interventricular septum; LA, left atrial; LAVi, left atrial volume index; LV, left ventricular; LVEDD, left ventricular end-diastolic diameter; LVEDVi, left ventricular end-diastolic volume index; LVEF, left ventricular ejection fraction; LVESVi, left ventricular end-systolic volume index; LVMi, left ventricular mass index; MAP, mean arterial pressure; MAPSE, mitral annular plane systolic excursion; MR, mitral regurgitation; PW, posterior wall; RVIT, right ventricular inﬂow tract; RWT, relative wall thickness; SBP, systolic blood pressure; SPAP, systolic pulmonary artery pressure; SVi, stroke volume index; TAPSE, tricuspid annular plane systolic excursion; TPRi, total peripheral resistance index; TR, tricuspid regurgitation.

	Android obesity (n=40)	Gynoid obesity (n=40)	Controls (n=40)	p-value
Conventional echoDoppler parameters				
IVS (mm)	12.5 ± 2.1	10.5 ± 2.2	9.0 ± 1.9	<0.001
PW (mm)	10.0 ± 1.3	8.8 ± 1.1	8.1 ± 1.2	<0.001
LVEDD (mm)	46.5 ± 3.7	46.6 ± 4.6	46.5 ± 3.1	0.99
RWT	0.43 ± 0.06	0.37 ± 0.06	0.35 ± 0.05	<0.001
LVMi (g/m^2^)	86.9 ± 17.4	76.5 ± 17.3	71.0 ± 12.0	<0.001
LV concentric remodeling (n, %)	19 (47.5)	5 (12.5)	6 (15.0)	<0.001
LV concentric hypertrophy (n, %)	4 (10.0)	1 (2.5)	1 (2.5)	0.21
LV eccentric hypertrophy (n, %)	2 (5.0)	8 (20.0)	1 (2.5)	0.01
Normal LV geometric pattern (n, %)	15 (37.5)	27 (60.0)	33 (82.5)	<0.001
LVEDVi (ml/m^2^)	36.1 ± 9.5	40.8 ± 8.2	40.1 ± 7.0	0.03
LVESVi (ml/m^2^)	12.1 ± 3.0	13.8 ± 3.3	13.8 ± 2.7	0.02
LVEF (%)	66.5 ± 2.3	66.2 ± 3.4	65.6 ± 4.0	0.47
MAPSE (mm)	17.1 ± 3.8	20.5 ± 2.1	20.2 ± 2.2	<0.001
E/A ratio	0.96 ± 0.33	1.08 ± 0.30	0.97 ± 0.30	0.16
First degree of LV diastolic dysfunction (n, %)	27 (67.5)	12 (30.0)	30 (75.0)	<0.001
E/average e’ ratio	10.0 ± 3.1	5.5 ± 2.7	7.4 ± 2.8	<0.001
LA A-P diameter (mm)	42.0 ± 5.1	39.3 ± 4.0	35.5 ± 4.2	<0.001
LA longitudinal diameter (mm)	54.0 ± 3.8	50.2 ± 4.9	47.3 ± 3.5	<0.001
LAVi (ml/m^2^)	30.2 ± 6.5	26.0 ± 6.8	30.6 ± 6.3	0.003
Mild-to-moderate MR (n, %)	8 (20.0)	6 (15.0)	5 (12.5)	0.64
Mild-to-moderate AR (n, %)	5 (12.5)	4 (10.0)	3 (7.5)	0.76
Mild-to-moderate TR (n, %)	15 (37.5)	11 (27.5)	8 (20.0)	0.22
RVIT (mm)	30.3 ± 2.5	30.5 ± 3.0	29.9 ± 3.2	0.64
TAPSE (mm)	23.7 ± 4.1	27.6 ± 4.1	26.6 ± 3.3	<0.001
SPAP (mmHg)	27.1 ± 4.3	26.6 ± 5.8	25.7 ± 3.9	0.41
Aortic root (mm)	32.7 ± 4.6	29.8 ± 2.8	29.6 ± 2.6	<0.001
Ascending aorta (mm)	33.0 ± 4.4	30.0 ± 3.9	28.9 ± 2.8	<0.001
Aortic arch (mm)	28.9 ± 3.5	26.7 ± 2.3	25.8 ± 2.0	<0.001
End-systolic EAT (mm)	8.5 ± 1.2	4.5 ± 1.3	4.2 ± 1.5	<0.001
Hemodynamic indices				
HR (bpm)	74.2 ± 13.7	72.7 ± 11.0	77.7 ± 12.5	0.19
SBP (mmHg)	128.4 ± 8.2	116.6 ± 8.4	118.3 ± 17.0	<0.001
ESP (mmHg)	115.6 ± 7.4	104.9 ± 7.5	106.5 ± 15.3	<0.001
DBP (mmHg)	80.2 ± 8.1	70.7 ± 7.4	73.3 ± 12.6	<0.001
MAP (mmHg)	96.3 ± 6.8	86.0 ± 6.7	88.3 ± 13.5	<0.001
SVi (ml/m^2^)	32.1 ± 4.7	38.1 ± 6.6	36.9 ± 6.0	<0.001
EaI (mmHg/ml/m^2^)	3.67 ± 0.59	2.83 ± 0.49	2.99 ± 0.75	<0.001
COi (l/min/m^2^)	2.38 ± 0.50	2.78 ± 0.73	2.84 ± 0.56	0.002
TPRi (dyne.sec/cm^5^)/m^2^	3387.1 ± 794.7	2612.2 ± 576.5	2597.1 ± 722.2	<0.001

On 2D-STE analysis, all bi-atrial and bi-ventricular strain and strain rate parameters measured in women with android obesity were significantly reduced in comparison to those with gynoid obesity and controls (all p <0.001). Secondly, all myocardial deformation indices measured in women with gynoid obesity were significantly increased in comparison to controls (Table 3).

**Table 3:** Myocardial strain and strain rate parameters assessed by 2D-STE analysis obtained in the two groups of 40 healthy women with “apple-shaped” and “pear-shaped” obesity and in the 40 controls, enrolled in the present study. Data are expressed as mean ± SD. Significant P values are in bold. 2D, two-dimensional; FWLSS, free wall longitudinal systolic strain; GCS, global circumferential strain; GCSR, global circumferential strain rate; GLS, global longitudinal strain; GLSR, global longitudinal strain rate; GRS, global radial strain; GRSR, global radial strain rate; GSA–, negative global atrial strain; GSA+, positive global atrial strain; GSR+, positive global strain rate; GSRE, global early-diastolic strain rate; GSRL, global late-diastolic strain rate; LA, left atrial; LV, left ventricular; RA, right atrial; RV, right ventricular; STE, speckle tracking echocardiography; TGSA, total global atrial strain.

Biatrial and biventricular strain and strain rate variables	Android obesity (n=40)	Gynoid obesity (n=40)	Controls (n=40)	p-value
LA-GSA+ (%)	19.7 ± 6.3	34.2 ± 4.6	29.8 ± 7.9	<0.001
LA-GSA- (%)	-6.3 ± 3.2	-10.3 ± 4.7	-8.5 ± 4.8	<0.001
LA-TGSA (%)	26.0 ± 5.9	44.5 ± 4.1	38.3 ± 9.8	<0.001
LA-GSR (s^-1^)	1.46 ± 0.49	1.99 ± 0.41	1.72 ± 0.46	<0.001
LA-GSRE (s^-1^)	-1.65 ± 0.58	-2.55 ± 0.38	-2.26 ± 0.72	<0.001
LA-GSRL (s^-1^)	-2.13 ± 0.58	-2.76 ± 0.31	-2.43 ± 0.56	<0.001
E/e’/LA-GSA+	0.56 ± 0.24	0.16 ± 0.09	0.25 ± 0.13	<0.001
LV-GLS (%)	-16.2 ± 3.4	-23.7 ± 2.6	-21.1 ± 1.6	<0.001
LV-GLSR (s^-1^)	-0.98 ± 0.16	-1.31 ± 0.25	-1.14 ± 0.08	<0.001
LV-GCS (%)	-19.2 ± 3.9	-28.3 ± 2.4	-24.5 ± 2.1	<0.001
LV-GCSR (s^-1^)	-1.31 ± 0.34	-1.97 ± 0.50	-1.58 ± 0.56	<0.001
LV-GRS (%)	25.7 ± 4.4	38.7 ± 2.0	36.5 ± 2.1	<0.001
LV-GRSR (s^-1^)	1.72 ± 0.51	2.95 ± 0.15	2.08 ± 0.41	<0.001
RA-GSA+ (%)	19.8 ± 6.3	27.3 ± 5.6	23.2 ± 5.7	<0.001
RA-GSA- (%)	-5.6 ± 4.1	-9.6 ± 2.8	-7.7 ± 3.4	<0.001
RA-TGSA (%)	25.4 ± 5.6	36.9 ± 6.9	30.9 ± 7.6	<0.001
RA-GSR (s^-1^)	1.52 ± 0.55	1.96 ± 0.33	1.71 ± 0.54	<0.001
RA-GSRE (s^-1^)	-1.65 ± 0.66	-2.25 ± 0.51	-1.95 ± 0.64	<0.001
RA-GSRL (s^-1^)	-2.02 ± 0.57	-2.66 ± 0.19	-2.36 ± 0.63	<0.001
RV-FWLSS (%)	-15.9 ± 2.6	-22.0 ± 1.9	-20.5 ± 3.6	<0.001
RV-GLS (%)	-15.2 ± 2.7	-21.1 ± 1.8	-19.1 ± 3.6	<0.001
RV-GLSR (s^-1^)	-1.01 ± 0.18	-1.83 ± 0.31	-1.56 ± 0.48	<0.001

Considering the accepted reference values reported in the literature [31], LV-GLS was reduced (less negative than -20%) in 26 women with android obesity (65% of total), in two women with gynoid obesity only (5%), and in nine controls without obesity (22.5%).

**Figure 1:**
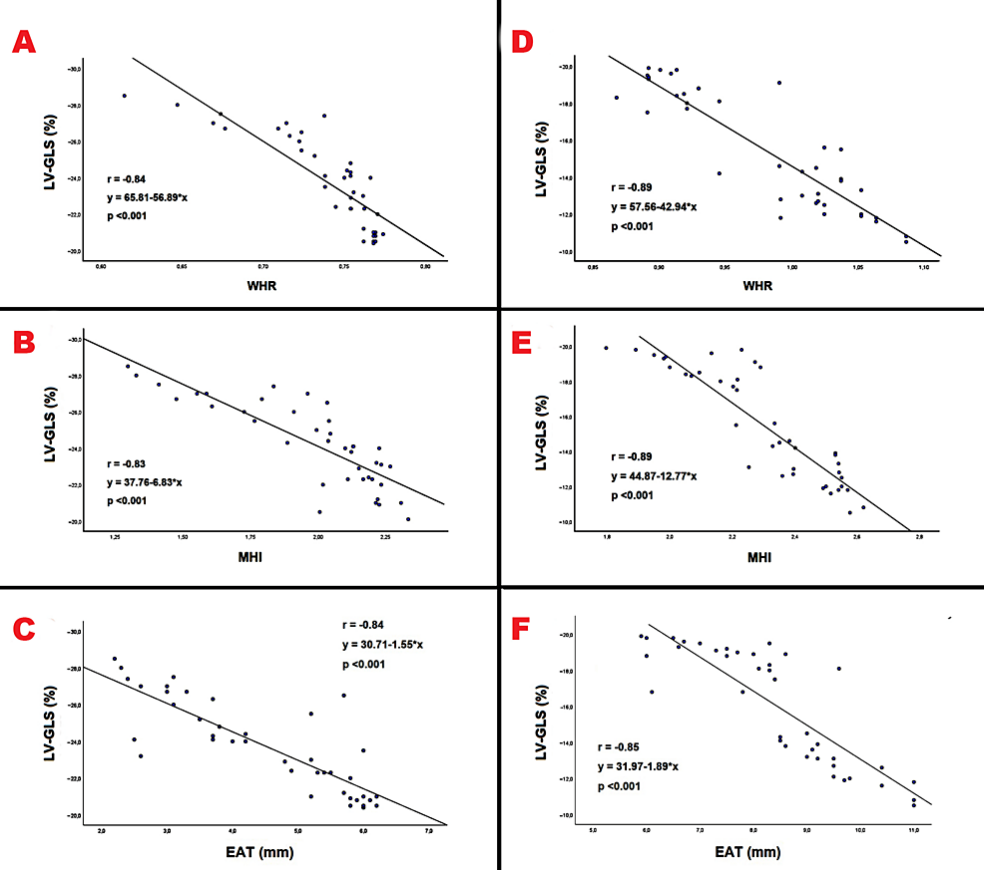
Correlation between LV-GLS and following anthropometrics: WHR, MHI, and EAT, evaluated separately in women with gynoid obesity (Panels A, B, and C) and in those with android obesity (panels D, E, and F), by using the Pearson’s correlation coefﬁcient. EAT, epicardial adipose tissue; GLS, global longitudinal strain; LV, left ventricular; MHI, modified Haller index; WHR, waist-to-hip ratio.

In the present study, we tested the hypothesis that the different magnitude of myocardial strain and strain rate indices observed in individuals with android obesity compared to those with gynoid obesity might be primarily related to a likely extrinsic cardiac compression and/or heart displacement exerted by anthropometric factors, such as WHR, MHI, and EAT. Accordingly, we evaluated the correlation between LV-GLS and all the above-mentioned anthropometrics, in both groups of women with android and gynoid obesity. A strong inverse correlation between LV-GLS and all main anthropometrics (WHR, MHI, and EAT) was demonstrated in both groups of obese women (Figure 1).

The higher were the WHR, the MHI and the EAT absolute values, the lower were the LV-GLS magnitude, likely due to an extrinsic thoracic compression and/or heart displacement synergically exerted by abdominal obesity, subcutaneous thoracic adiposity and intrathoracic visceral adiposity on cardiac chambers. On the other hand, the larger myocardial deformation observed in healthy women with gynoid obesity than controls could be attributed to a more spheroidal transversal thoracic shape in such women, with consequent reduced extrinsic thoracic compression on cardiac chambers and less heart displacement in comparison to controls. In addition, among healthy controls, those women with a mild impairment in LV-GLS (22.5% of total) had a narrow A-P thoracic diameter (<13 cm), which could be responsible for a reduced longitudinal myocardial deformation in such women.

**Figure 2:**
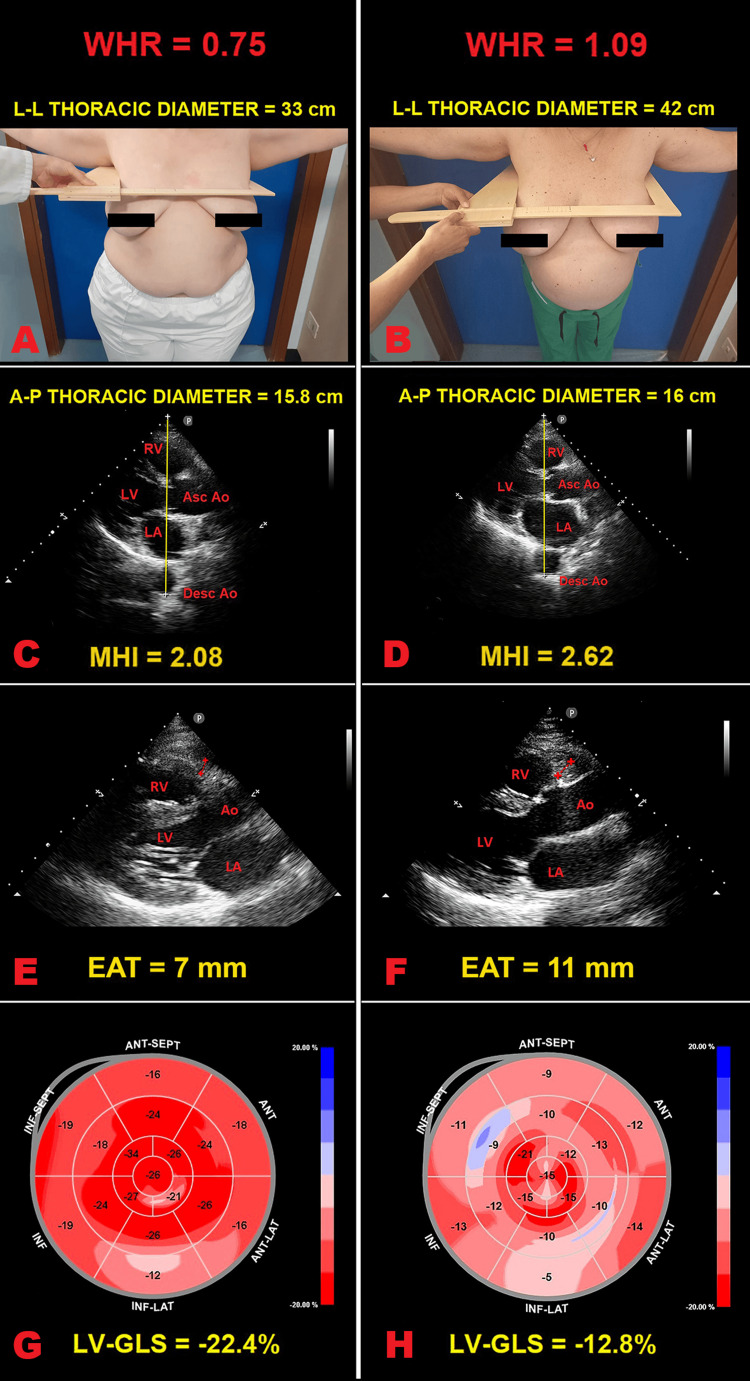
Examples of L-L thoracic diameter (panels A and B), A-P thoracic diameter (panels C and D), EAT (panels E and F), and LV-GLS bull’s eye plot (panels G and H) assessment in healthy women with gynoid obesity and in a healthy woman with android obesity respectively, enrolled in the present study. Panels A and B: The maximum L-L external thoracic diameter, measured with the obese woman in the standing position and with open arms, by using a rigid ruler in centimeters coupled to a level (the measuring device), placed at the distal third of the sternum, in the point of maximum depression of the sternum. Panels C and D: The minimum A-P internal thoracic diameter, obtained with the obese women in left-lateral decubitus position, during the conventional transthoracic echocardiography, by placing a 2.5 mHz transducer near the sternum in the left third or fourth intercostal space, to obtain a parasternal long-axis view, and measuring the distance between the true apex of the sector (the point of entry of ultrasound) and the anterior surface of the vertebral body. The vertebral body is identified by using, as a reference point, the posterior wall of the descending thoracic aorta, visualized behind the left atrium. Panels E and F: End-systolic EAT thickness, measured on the free wall of the right ventricle, from the parasternal long-axis view on standard 2D-TTE. Panels G and H: Examples of LV-GLS bull’s eye plot, assessed by 2D-STE analysis. The healthy woman with “pear-shaped” obesity (WHR = 0.75) showed an uniformly red pattern of the bull’s eye plot, representing a normal range in strain values (Panel G), whereas the healthy woman with android obesity (WHR = 1.09) showed a significant impairment in LV-GLS magnitude (Panel H). 2D, two-dimensional; Ao, aorta; A-P, antero-posterior; Asc, ascending; Desc, descending; EAT, epicardial adipose tissue; GLS, global longitudinal strain; LA, left atrium; L-L, latero-lateral; LV, left ventricle/ventricular; MHI, modified Haller index; RV, right ventricle; STE, speckle tracking echocardiography; TTE, transthoracic echocardiography; WHR, waist-to-hip ratio.

Figure 2 illustrates examples of L-L thoracic diameter (panels A and B), A-P thoracic diameter (panels C and D), EAT (panels E and F) and LV-GLS bull’s eye plot (panels G and H) assessment in healthy women with gynoid obesity and in a healthy woman with android obesity, respectively, enrolled in the present study.

Univariate logistic regression analysis revealed that the WHR (OR 1.58, 95%CI 1.22-2.03, p <0.001) and LVMi (OR 1.09, 95%CI 1.02-1.16, p = 0.006) were independently correlated with LV-GLS impairment (defined as an absolute value less negative than -20%) in women with android obesity. On multivariate logistic regression analysis, the WHR maintained a statistically significant association with the above-mentioned outcome (OR 1.68, 95%CI 1.14-2.48, p = 0.009) (Table 4).

**Table 4:** Univariate and multivariate logistic regression analysis performed to evaluate the effect of the main demographic, anthropometric, glycometabolic, and conventional echocardiographic variables on LV-GLS impairment (deﬁned as an absolute value less negative than -20%) in women with android obesity. Signiﬁcant p-values are in bold. HOMA-IR, Homeostasis Model Assessment for Insulin Resistance; LVMi, left ventricular mass index; WHR, waist-to-hip ratio.

	Univariate Logistic Regression Analysis			Multivariate Logistic Regression Analysis		
Variables	OR	95% CI	p-value	OR	95% CI	p-value
Age (yrs)	1.04	0.98-1.09	0.18			
WHR (x 0.01 increase)	1.58	1.22-2.03	<0.001	1.68	1.14-2.48	0.009
HOMA-IR	1.12	0.84-1.48	0.44			
LVMi (g/m^2^)	1.09	1.02-1.16	0.006	1.07	0.96-1.20	0.19

**Figure 3:**
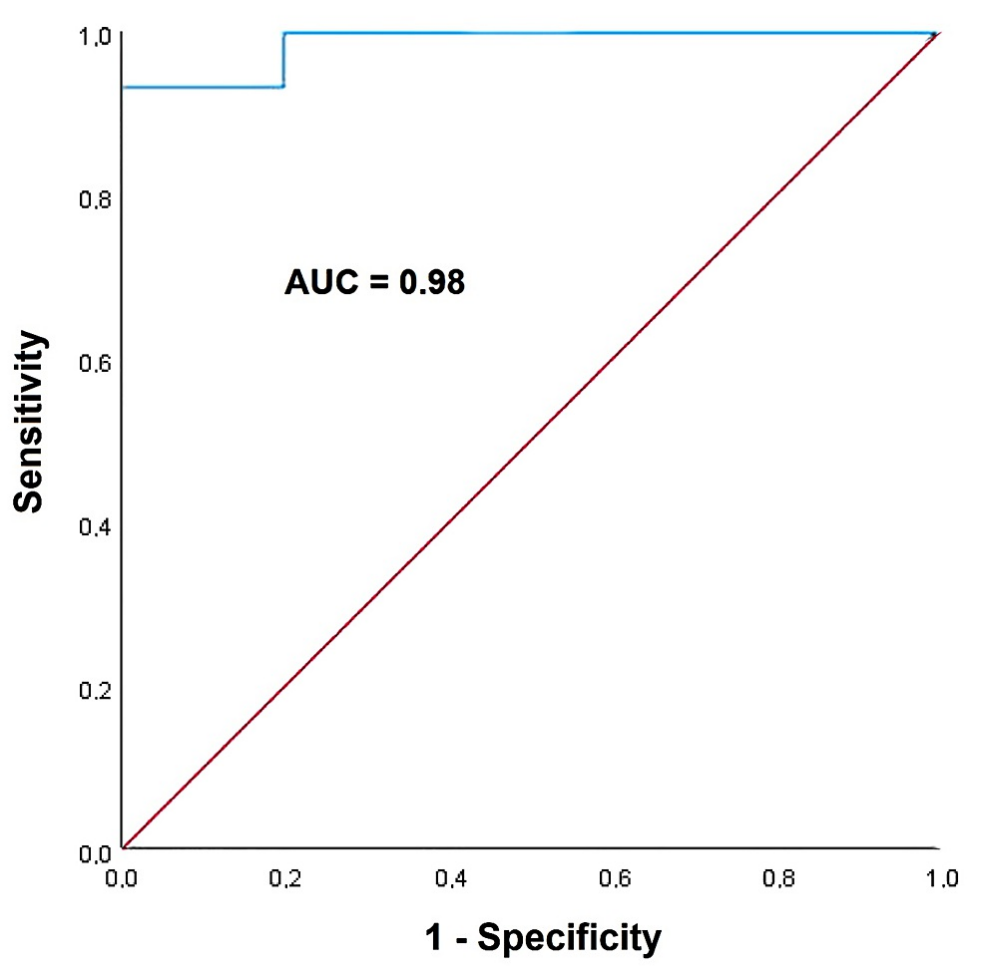
ROC curve drawn to assess the sensitivity and the speciﬁcity of WHR for predicting an absolute value of LV-GLS less negative than -20%, in healthy women with android obesity. AUC, area under the curve; GLS, global longitudinal strain; LV, left ventricular; ROC, receiver operating characteristic; WHR, waist-to-hip ratio.

ROC curve analysis showed that a WHR value ≥1.01 had 93% sensitivity and 100% specificity for detecting LV-GLS impairment in women with android obesity (AUC = 0.98; 95%CI 0.96-1.00) (Figure 3).

Measurement variability

Intra-observer and inter-observer agreement between the raters, expressed as ICCs with 95% CIs, for LV-GLS assessment, was 0.89 (95% CI 0.64-0.97) and 0.83 (95%CI 0.45-0.95), respectively.

## Discussion

The present study that retrospectively analyzed two cohorts of women with opposite phenotypes of obesity demonstrated the important influence exerted by anthropometrics in determining an early impairment in myocardial strain parameters in healthy women with obesity and without overt heart disease. Notably, the greater were the WHR, the MHI and the EAT absolute values, the lower was the LV-GLS magnitude, as observed in healthy women with android obesity; the smaller were the WHR, the MHI and the EAT absolute values, the larger was the LV-GLS magnitude, as detected in women with gynoid obesity. The WHR showed a strong correlation with LV-GLS impairment in healthy women with android obesity, independent of age, glycometabolic status, and LVMi. A WHR value ≥1.01 showed the greatest sensitivity and specificity for predicting an LV-GLS value less negative than -20% in healthy women with android obesity. Differently from healthy women with “apple-shaped” obesity, those with “pear-shaped” obesity were found with normal cardiac chamber cavity sizes, normal systolic and diastolic function on conventional 2D-TTE, and supernormal myocardial strain parameters on 2D-STE analysis. These findings were likely related to a more spheroidal transversal chest conformation with less heart displacement and reduced extrinsic cardiac compression exerted by abdominal and thoracic fat accumulation, in women with gynoid obesity. As far as we know, this is the first study that demonstrated the independent effect of thoracoabdominal adiposity on myocardial strain parameters in healthy women with opposite obesity phenotypes.

Consistent with previous 2D-STE studies conducted in individuals with obesity [3-5], our results confirmed that central obesity impairs both LV and RV mechanics. The alteration in myocardial strain indices observed in individuals with abdominal obesity was ascribed by previous authors to several cardiometabolic factors, such as visceral fat area accumulation [5], increased blood pressure values [32], elevated waist circumference [33], high serum levels of fasting glucose [34], insulin resistance [35], and a chronic low-grade inflammatory state characterized by increased production of proinflammatory cytokines [36,37]. All these stimuli may result in impaired myocardial energetics, myocyte apoptosis, and increased fibrosis, thus synergically contributing to an early reduction in LV-GLS [38].

Differently from the above-mentioned studies, we hypothesized that the subclinical myocardial dysfunction early detected by 2D-STE analysis in healthy women with android obesity might be, at least in part, related to an extrinsic mechanical compression on cardiac chambers and/or heart displacement exerted by the combination of abdominal obesity, subcutaneous thoracic adiposity and increased amount of epicardial adipose tissue around the heart. With this regard, we demonstrated a strong inverse correlation between LV-GLS magnitude and all anthropometrics (WHR, MHI, and EAT). Among these anthropometrics, the WHR was the only independent predictor of reduced LV-GLS (less negative than -20%), in women with android obesity.

The results of our study also confirmed that lower-body obesity was inversely associated with hyperinsulinemia, dyslipidemia, and LV-GLS impairment in healthy individuals with obesity [6-8]. These findings could be explained not only by a less inflammatory profile of lower-body adipose tissue which confers resistance against obesity-associated metabolic complications [7,8], but also by a significantly lower abdominal adiposity, a significantly smaller L-L thoracic diameter with more spheroidal transversal chest conformation, and finally significantly lower intrathoracic visceral adipose accumulation. Accordingly, both LV and RV cardiac mechanics of healthy women with gynoid obesity could be less influenced by anthropometrics, leading to apparently much more efficient strain imaging measurements than those of healthy women with “apple-shaped” obesity and controls without obesity. Indeed, consistent with what was reported by our study group in healthy individuals with pectus excavatum or even a mild degree of anterior chest wall deformity [39], a number of healthy individuals without obesity and without overt heart disease may be found with a certain degree of LV-GLS impairment due to extrinsic thoracic compression and/or heart displacement, with consequent apparently reduced kinetics of basal myocardial segments, in absence of any intrinsic myocardial dysfunction.

The main implications of our findings are that a combined WHR, MHI, and EAT assessment should be implemented in clinical practice, in order to better identify, among women with different phenotypes of obesity, those with increased probability of being diagnosed with early subclinical myocardial dysfunction. Notably, detection of a concomitant increase in WHR, MHI, and EAT might predict an LV-GLS value less negative than -20% on 2D-STE analysis in healthy women with obesity and without overt heart disease.

Given that LV-GLS impairment has been demonstrated to be a powerful and independent predictor of cardiovascular outcome even in the general population [40] and that obesity is a risk factor for the development of heart failure with preserved ejection fraction [41], healthy women with “apple-shaped” obesity should undergo early treatment strategies. In particular, these women should undergo more aggressive and timely targeted non-pharmacological interventions, including weight loss intervention (diet and increased physical activity) and, if necessary, intensive pharmacological management of glucose intolerance, hypertension, and dyslipidemia. In addition, during the last years, innovative treatments, such as deep Transcranial Magnetic Stimulation (dTMS), have demonstrated long-term beneficial effects in reducing food cravings and body weight in individuals with obesity [42].

Limitations of the study

The main limitations of the present study were its retrospective nature, the monocentric design, and the small sample size of individuals with obesity enrolled. However, a statistical power analysis was performed for this prospective study and the sample size was adequate to detect a statistically significant difference with regard to main functional echocardiographic parameters under investigation.

Biochemical parameters did not include proinflammatory cytokines, such as C-reactive protein, tumor necrosis factor-alpha, and interleukin-6. Moreover, we did not measure serum adiponectin levels, which were inversely associated with LV hypertrophy and diastolic dysfunction in previous population-based studies [43,44].

Speckle-tracking analysis suffers from some limitations, such as inter-vendor variability, dependence on good image quality, and temporal stability of tracking patterns [45].

Even if the same software employed for the ventricular strain analysis was used for the assessment of biatrial mechanics, it was able to clearly deﬁne both LA and RA deformation throughout the cardiac cycle and to obtain a rapid assessment of both atrial and ventricular myocardial strain parameters.

## Conclusions

The WHR is independently associated with reduced LV-GLS magnitude in healthy women with android obesity, independent of age, glycometabolic status, and LV size. Further studies are needed to evaluate if more aggressive and timely targeted non-pharmacological and/or pharmacological interventions might improve LV-GLS by reducing the WHR.
